# The mitochondrial carrier SLC25A10 regulates cancer cell growth

**DOI:** 10.18632/oncotarget.3375

**Published:** 2015-03-03

**Authors:** Xiaoshan Zhou, João A. Paredes, Shuba Krishnan, Sophie Curbo, Anna Karlsson

**Affiliations:** ^1^ Division of Clinical Microbiology, Department of Laboratory Medicine, Karolinska Institute, Karolinska University Hospital Huddinge, 141 86 Stockholm, Sweden

**Keywords:** oxidative stress, redox homeostasis, ROS, SLC25A10

## Abstract

Dysregulation of cell metabolism is critical for the growth properties of cancer cells. The purpose of this study was to understand the role of substrate transport across the mitochondrial membrane to sustain the metabolic shift and redox defense in cancer cells. Mitochondrial carrier *SLC25A10* is up-regulated in a variety of tumors and is involved in regulating intracellular levels of reactive oxygen species. We show that knockdown of *SLC25A10* in A549 cells changed the growth properties to a less malignant phenotype and casued increased glutamine dependency and sensitivity to oxidative stress. The metabolic alteration was linked to an energy metabolic shift from glycolysis to mitochondrial oxidative phosphorylation illustrated by increased expression of glutamate dehydrogenase, decreased expression of lactate dehydrogenase due to down-regulation of hypoxia inducible factor 1α. We identified effects on NADPH production linked to the growth changes observed in *SLC25A10* knockdown cells, demonstrated by decreased NADPH production in cells deprived of glutamine. The contribution of *SLC25A10* to reprogram cell metabolism and to regulate cell growth suggests *SLC25A10* as a novel target for anti-cancer strategies.

## INTRODUCTION

The solute carrier 25 (SLC25) family of nuclear-encoded transporters in the mitochondrial inner membrane are involved in numerous metabolic pathways. Based on substrate specificity these carriers are divided into subfamilies with different functional characteristics [1]. The major role of SLC25 family member 10 (SLC25A10), also known as dicarboxylate carrier, is to transport the dicarboxylate substrates malate and succinate, out of the mitochondria in exchange for phosphate, sulfate, and thiosulfate, thus supplying substrates for gluconeogenesis, urea synthesis, and sulfur metabolism [2]. By doing that, it maintains the balance of the TCA cycle intermediates between the mitochondria and the cytosol [3, 4]. Suppression of *SLC25A10* has been shown to reduce the citrate transport from mitochondria to the cytosol and inhibit the *de novo* fatty acid synthesis in hepatocytes [5]. This carrier was also shown to participate in glucose-stimulated insulin secretion through pyruvate-cycling [6]. Furthermore, the SLC25A10 carrier has been linked to reactive oxygen species (ROS) production with hyperpolarization of mitochondria and increased ROS levels when *SLC25A10* was over expressed in cultured cells [7]. Altogether, the evidence suggests that SLC25A10 participates in both energy metabolism and redox homeostasis. Interestingly, increased *SLC25A10* expression has been demonstrated in a variety of tumors although the exact role of *SLC25A10* in tumor cells is not known [8, 9]. In addition to SLC25A10, other mitochondrial carriers of the SLC25 family are also involved in cancer [10–12]. Altered energy metabolism and redox homeostasis is frequently identified in tumor cells [13, 14]. A result of these metabolic changes is that the production of NADPH and glutathione (GSH), both important anti-oxidants, is modulated in cancer cells [15]. NADPH is crucial for the biosynthesis of macromolecules as well as to defend cells from oxidative stress and GSH is the major antioxidant produced by cells. The production of NADPH has been suggested to be of special importance for cancer cell metabolism [15]. In proliferating cells NADPH is mainly produced through the pentose phosphate pathway (PPP), but important contributions to NADPH production is also through the reaction converting malate to pyruvate [16].

Based on the evidence of altered expression of *SLC25A10* in tumor cells we were interested in the role of *SLC25A10* to maintain the growth properties of tumor cells in culture. Here, we investigated the effects of decreased expression of *SLC25A10* on cell growth, NADPH production and redox homeostasis in the non-small cell lung cancer (NSCLC) cell line A549. Overall our study proposes the importance of a functional SLC25A10 carrier to maintain properties of cancer cells, such as NADPH production independent of the PPP pathway. Gene expression analysis of key regulatory enzymes involved in cell metabolism and cell redox homeostasis provide evidence for a metabolic shift from aerobic glycolysis to mitochondrial oxidative phosphorylation in confluent *SLC25A10* knockdown cells.

In conclusion, our data demonstrate that the SLC25A10 carrier plays an important role in regulating redox homeostasis to protect confluent cells against oxidative stress. We propose SLC25A10 as a novel target for anti-tumor compound development with the aim to reprogram cell metabolism, compromise cell growth and increase sensitivity to the important anticancer drug cisplatin.

## RESULTS

### Establishment and characterization of a stable *SLC25A10* knockdown cell line

**Figure 1:**
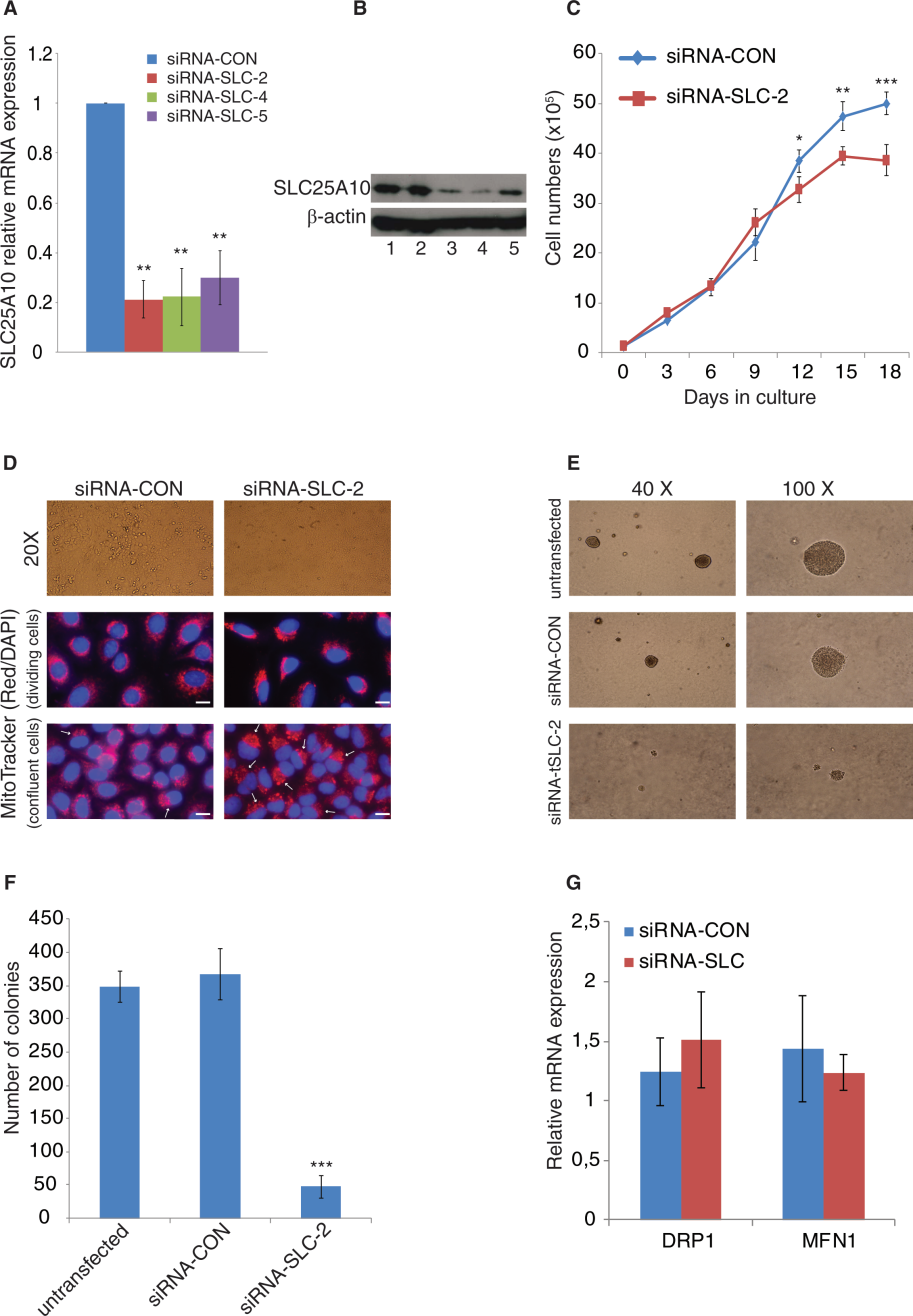
Growth behavior and morphology changes of SLC25A10 knockdown cells Establishment of stable SLC25A10 knockdown cell lines with specific siRNA expression in A549 cell line. **(A)** SLC25A10 gene expression was analysed by real-time qPCR in different clones (siRNA-SLC-2, siRNA-SLC-4 and siRNA-SLC-5). **(B)** SLC25A10 protein expression in A549 cells; 1, untransfected cells; 2, siRNA-CON cells; 3, siRNA-SLC-2; 4, siRNA-SLC-5. **(C)** Growth curve of siRNA-SLC-2 cell line, data is represented as mean ± SD. **(D)** Cell morphology change (magnification 20X) and mitochondrial staining. Cells with altered mitochondria are indicated with arrows and scale bar represents 20 μm **(E)** Sizes of colonies. **(F)** Numbers of colonies. This experiment was repeated 3 times independently, data is presented as mean ± SD, *** represents *p* < 0, 001. **(G)** Mitochondrial DRP1 and MFN1 gene expression levels.

Stable knockdown A549 NSCLC cell lines (siRNA-SLC −2, −4 and −5) with more than 75% reduction of *SLC25A10* mRNA were established (Figure 1A). The SLC25A10 protein levels decreased by 73%, 80% and 37% in siRNA-SLC −2, −4 and −5 compared to the siRNA-CON and untransfected cells (Figure 1B). The down-regulation of *SLC25A10* did not affect the doubling time of both cell types, however, after reaching confluency the siRNA-CON cells had a higher proliferation rate than the siRNA-SLC cells (Figure 1C). The siRNA-SLC cells grew in a monolayer manner and displayed decreased ability to form cell islands compared to untransfected A549 or siRNA-CON cells (Figure 1D). Moreover, the size of the siRNA-SLC cells was smaller than the size of the siRNA-CON cells (Figure 1D). Since siRNA-SLC cells grew in an even monolayer, soft agar experiments were performed to compare the ability of anchorage-independent growth of *SLC25A10* knockdown cells with untransfected and mock control cells. The sizes of colonies from siRNA-SLC were small compared to the colonies formed by untransfected or siRNA-CON cells (Figure 1E) and in addition the number of colonies formed by the siRNA-SLC cells were significantly lower than in untransfected or siRNA-CON cells (Figure 1F).

The role of SLC25A10 for maintaining mitochondrial morphology was investigated. MitoTracker staining shows a difference in that localization of the cell nucleus with a polarized pattern of mitochondria at a single side of the cell nucleus, and not surrounding the nucleus, as compared to siRNA-CON (Figure 1D) especially in confluent cells with 89% and 12% positive cells in siRNA-SLC and siRNA-CON cells respectively (*p* < 0, 001). Meanwhile, the gene expression of two genes important for mitochondrial morphology, dynamin-related protein 1 (*DRP1*) and mitofusin 1 (MFN1), which control mitochondrial fusion and fission respectively, were not significantly changed as determined by real-time PCR (Figure 1G).

### Increased dependency on glutamine in confluent siRNA-SLC cells

**Figure 2:**
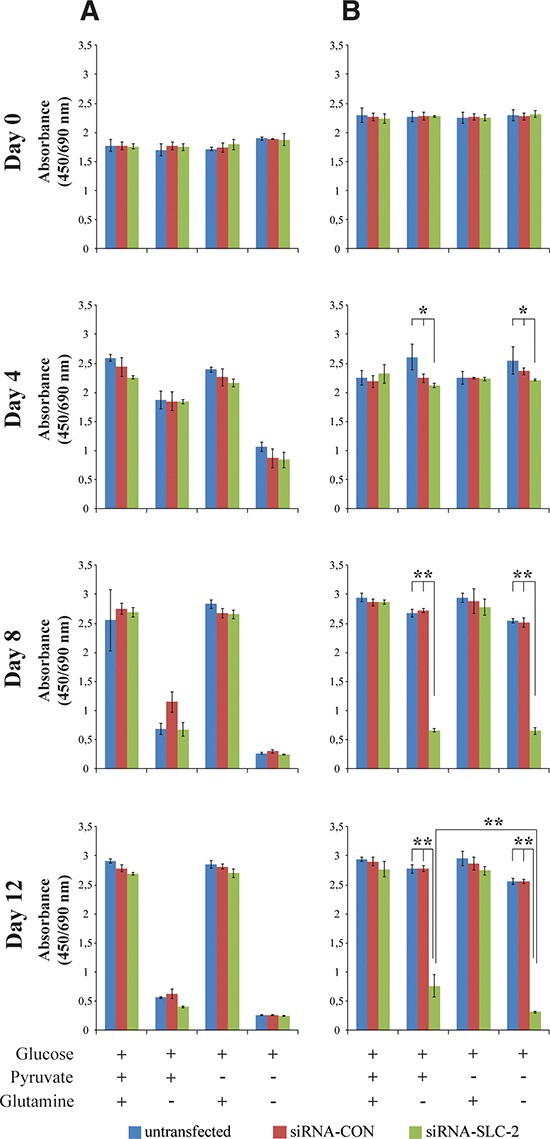
Glutamine sensitivity changes in proliferating and confluent SLC25A10 knockdown A549 cells **(A)** Proliferating cells **(B)** Confluent cells. Glucose, glutamine and pyruvate concentrations were 25 mM, 2 mM and 1 mM respectively. Data represents absorbance at (450/690 nm). The experiment was repeated 3 times, and all data are presented as mean ± SD, ** represents *p* < 0.01.

SLC25A10 transports malate and succinate from the mitochondria across the mitochondrial inner membrane. In the mitochondria malate and succinate are converted to oxaloacetate, which is further transformed into aspartate by an addition of an amino radical supplied by glutamate. To investigate the glutamine dependency of the cells glutamine deprivation experiments were performed at two cell densities since cells have different growth properties at different growth stages: active proliferating cells and confluent cells. In proliferating cells, both siRNA-SLC and untransfected or siRNA-CON cells were sensitive to glutamine deprivation (Figure 2A). There was no significant difference between these cell lines cultured in medium without glutamine and pyruvate. For the confluent cells, reducing the expression of the carrier led to increased sensitivity to glutamine deprivation (Figure 2B). While confluent untransfected and siRNA-CON cells were resistant to glutamine withdrawal, confluent siRNA-SLC cells were sensitive to glutamine deprivation. Pyruvate could partially rescue both the actively proliferating and the confluent cells. These results were also confirmed with other A549 *SLC25A10* knockdown cell lines (Supplementary Figure S1) and Hela SLC25A10 knockdown cell lines (Supplementary Figure S2 A and S2 B).

### NADPH levels and ROS production in siRNA-SLC cells

**Figure 3:**
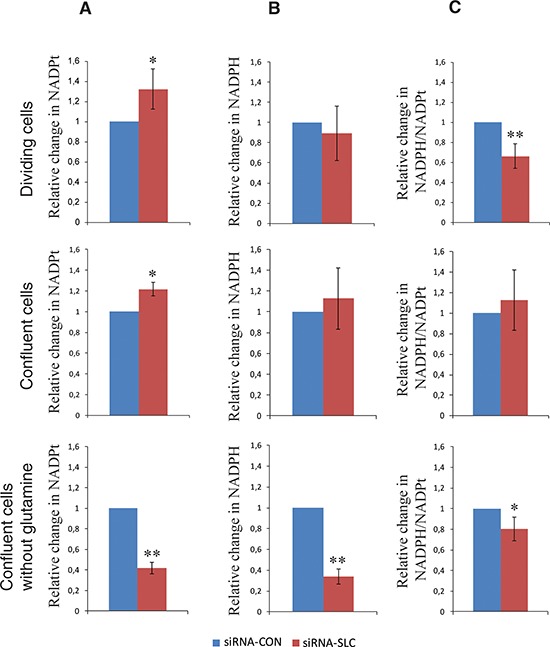
Total intracellular NADPt, NADPH levels and NADPH/NADPt ratios in SLC25A10 knockdown A549 cells at different growth situations **(A)** NADPt levels in dividing, confluent and confluent cells with glutamine starvation; **(B)** NADPH levels in dividing, confluent and confluent cells with glutamine starvation and **(C)** NADPH/NADPt ratios in dividing, confluent and confluent cells with glutamine starvation. Data represented as relative change of NADPt, NADPH and NADPH/NADPt in siRNA-SLC cells compared to siRNA-CON cells. All experiments are repeated 3 times independently, all data are presented as mean ± SD. * represents *p* < 0.05, ** represents *p* < 0.01.

To understand the mechanisms underlying the phenotype of siRNA-SLC cells, the NADPH levels were determined in dividing and confluent cells grown in complete media and in confluent cells also after 4 days of glutamine starvation. Interestingly, the total NADP increased by 20% in siRNA-SLC cells compared to siRNA-CON cells in both dividing and resting cells (Figure 3A). A slight decrease of NADPH in dividing siRNA-SLC cells and a slight increase of NADPH in resting siRNA-SLC cells (Figure 3B) resulted in a significant difference of the ratio of NADPH/total NADP in dividing cells although in resting cells the ratio between NADPH and total NADP was not altered between siRNA-SLC and siRNA-CON cells (Figure 3C). In cells grown without glutamine the difference between siRNA-SLC cells and siRNA-CON cells was pronounced. Both the amount of total NADP (Figure 3A) and NADPH (Figure 3B) were significantly decreased in the siRNA-SLC cells.

**Figure 4:**
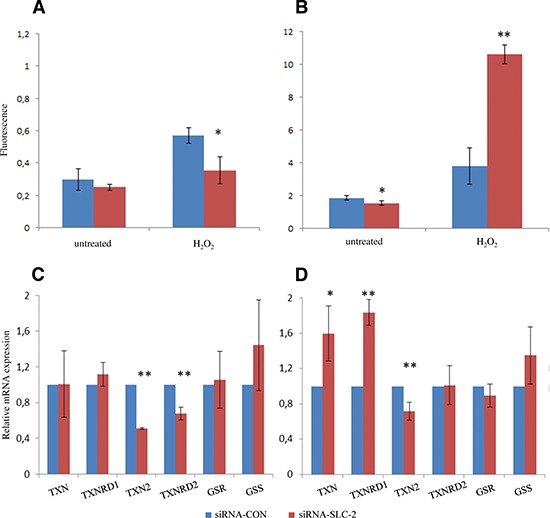
ROS and relevant genes expression level changes in SLC25A10 knockdown A549 cells **(A)** ROS levels of untreated and H_2_O_2_-treated dividing and **(B)** confluent cells were determined by fluorescence assay. Thioredoxins, thioredoxin reductases, glutathione and glutathione reductase genes expression was examined by real-time PCR in **(C)** dividing cells and **(D)** confluent cells. All experiments were repeated 3 times independently. * represents *p* < 0.05, ** represents *p* < 0.01.

Next, we measured the ROS levels in dividing and confluent cells. There was no difference in the level of ROS in untreated proliferating siRNA-SLC and siRNA-CON cells (Figure 4A). Confluent siRNA-SLC cells had significantly lower basal ROS level compared to siRNA-CON cells (Figure 4B). However, the proliferating and confluent siRNA-SLC cells responded differently to H_2_O_2_ challenges. The proliferating siRNA-SLC cells produced lower amounts of ROS than the siRNA-CON cells (Figure 4A), while the confluent siRNA-SLC cells produced significantly higher amounts of ROS (*p* < 0.01) than the confluent siRNA-CON cells (Figure 4B).

The thioredoxin system and the glutathione system are two of the major antioxidant systems in the mitochondrial matrix. To study if these systems were involved in regulation of the redox homeostasis linked to down-regulation of *SLC25A10*, the mRNA expression of glutathione synthase (*GSS*), glutathione reductase (*GSR*), thioredoxin 1 (*TXN*), thioredoxin 2 (*TXN2*) and the corresponding thioredoxin reductases 1 and 2 (*TXNRD1* and *TXNRD2*) were analyzed with real-time PCR. The results showed that the gene expression of *TXN2* and *TXNRD2* were significantly decreased in proliferating siRNA-SLC cells compared to siRNA-CON cells (*p* < 0.01) (Figure 4C). In confluent siRNA-SLC cells the gene expression of *TXN2* was also significantly down-regulated (*p* < 0.01), but the gene expression of *TXNRD2* was unaltered (Figure 4D). While *TXN* and *TXNRD1* were not changed in proliferating siRNA-SLC cells, the genes were significantly up-regulated in confluent siRNA-SLC cells (*p* < 0.05 and *p* < 0.01 respectively) (Figure 4D). No statistically significant difference was detected in the *GSS* and *GSR* gene expression between siRNA-SLC and siRNA-CON cells in neither proliferating nor confluent phases (Figure 4C and 4D).

### Changes in expression levels of metabolic regulatory enzymes

**Figure 5:**
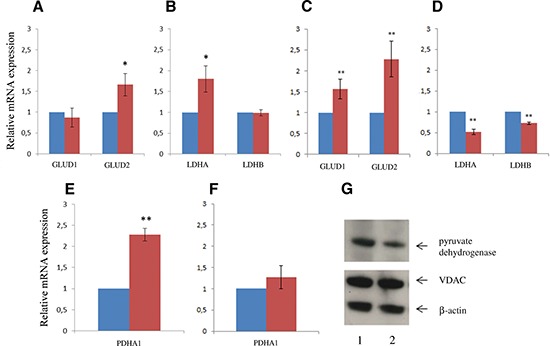
Key metabolic enzymes expression profile changes in proliferating and confluent SLC25A10 knockdown cells The mRNA levels of glutamine dehydrogenase, lactate dehydrogenase and pyruvate dehydrogenase were determined with real-time PCR in **(A, C, E)** dividing cells and **(B, D, F)** confluent cells. Three independent measurements were performed. Data presented as relative changes (mean ± SD) compared to siRNA-CON, ** represents *p* < 0.01. **(G)** Phosphorylated form of pyruvate dehydrogenase and mitochondrial VDAC protein levels were analyzed with Western-blot. β-actin was used as a loading control. 1, siRNA-CON; 2, siRNA-SLC-2.

Since SLC25A10 mainly transports dicarboxylates, genes involved in energy metabolism were also investigated. In dividing cells, the gene expression of glutamate dehydrogenase 2 (*GLUD2*) and lactate dehydrogenase A (*LDHA*) were significantly increased in siRNA-SLC cells (*p* < 0.05) (Figure 5A and 5B). In confluent cells, the gene expression of both glutamate dehydrogenase 1 and 2 (*GLUD1* and *GLUD2*) were also increased in siRNA-SLC cells (*p* < 0.01), whereas the gene expression of lactate dehydrogenase A and B (*LDHA* and *LDHB*) were decreased (*p* < 0.01) (Figure 5C and 5D). The transcript levels of pyruvate dehydrogenase A (*PDHA1*) were increased in proliferating siRNA-SLC cells (*p* < 0.01) (Figure 5E) but were similar in confluent siRNA-CON and siRNA-SLC cells (Figure 5F). However, the levels of the phosphorylated form of the pyruvate dehydrogenase protein was found to be decreased by 30–60% in confluent siRNA-SLC cells, when analyzed using a specific antibody against the phosphorylated site (ser 293) (Figure 5G).

**Figure 6:**
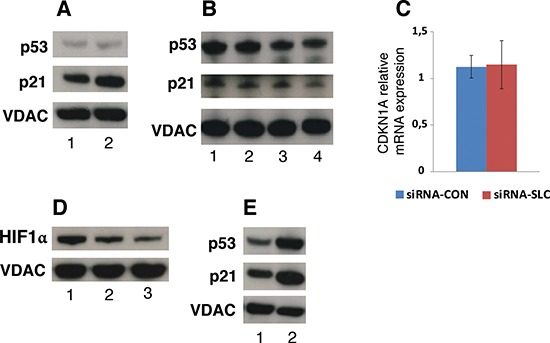
p21 and HIF-1α protein levels in SLC25A10 knockdown cells **(A)** p53 and p21 protein levels were analyzed with Western blot. 1, siRNA-CON; 2, siRNA-SLC-2. **(B)** p53 and p21 protein levels in untreated A549 cells cultured in conditioned DMEM media. 1, complete DMEM; 2, complete DMEM without pyruvate; 3, complete DMEM without glutamine; 4: complete DMEM without glutamine and pyruvate. VDAC was used as a loading control for these Western blots. **(C)** CDKN1A (p21) mRNA level in siRNA-SLC-2 cell line. This experiment was repeated 3 times independently. **(D)** HIF-1α protein levels detected by Western blot 1, siRNA-CON; 2, siRNA-SLC-2; 3, siRNA-SLC-5. **(E)** p53 and p21 protein levels in untransfected A549 cells treated with cisplatin. 1, control; 2, cisplatin.

To further explore signaling pathways that could be affected by down-regulation of *SLC25A10* the p53 protein expression was analyzed. Western blot showed that the p53 protein level was not significantly changed in siRNA-SLC cells compared to siRNA-CON cells, whereas the level of the p53 downstream target protein p21 was decreased in the siRNA-SLC cells (Figure 6A). To investigate if the down-regulation of p21 was linked to mitochondrial TCA intermediate disturbances caused by the *SLC25A10* knockdown, untransfected A549 cells were treated with media with or without glutamine or pyruvate. The p53 levels did not change in samples from cultures with glutamine, but decreased slightly in samples from cultures without glutamine and pyruvate (Figure 6B). Interestingly, the gene expression levels of p21 also slightly decreased in samples cultured without both glutamine and pyruvate (40–50%) (Figure 6B). The down-regulation of the p21 protein level occurred post-translationally, since the mRNA level was not changed (Figure 6C). Protein levels of hypoxia inducible factor 1α (HIF-1α), a key regulator of glycolysis in tumor cells, were also measured and showed to be decreased by 40% in siRNA-SLC cells (Figure 6D). In addition, the effect of the anticancer drug cisplatin on p21 expression was investigated. Both p53 and p21 were up-regulated upon cisplatin treatment (Figure 6E).

### Increased sensitivity to the anti-cancer drug cisplatin

Finally, to investigate the significance of *SLC25A10* for the activity of anti-cancer compounds, the sensitivity of cisplatin and 2′, 2-difluoro-deoxycytidine (dFdC) were determined (Table 1). The siRNA-SLC cells had lower IC_50_ for cisplatin compared with the siRNA-CON cells. There was, however, no difference in the sensitivity to dFdC between the cells investigated.

**Table 1:** Comparison of the sensitivity (IC_50_) of siRNA-CON and siRNA-SLC-2 cell lines to anticancer compounds (*n* = 3). CI, confidence interval

siRNA-CON			siRNA-SLC2		
IC_50_ [μM]					
Compound	Mean	95% CI	Mean	95% CI	*p*-value
cisplatin	18.3	15.8–21.2	9.8	8.1–11.7	< 0.0001
dFdC	0.053	0.017–0.161	0.035	0.007–0.168	0.63

## DISCUSSION

Metabolic modulation of cancer cells, as a way of therapeutically eliminate them, is of great interest in the field of cancer research. This work suggests that the mitochondrial carrier SLC25A10 is of importance for the properties that characterize cancer cells and hypothetically could be used as a target for that kind of regulation. In particular, our data show that confluent A549 cells with down-regulated *SLC25A10* have altered growth behavior and decreased ability to respond to oxidative stress, especially in resting cells. This change of redox homeostasis was linked to an energy metabolic shift from glycolysis to mitochondrial oxidative phosphorylation. In line with our findings is a recent study in which the SLC25A10 carrier was shown to be linked to ROS production in that *SLC25A10* was one of the target genes of the BACH1 transcription factor [17]. BACH1 is a heme-binding transcription factor and its target genes are involved in oxidative stress response and in control of the cell cycle. The substrates of the SLC25A10 carrier, malate and succinate, are linked to NADPH synthesis and regulation of cell metabolism, respectively. Malate is the substrate for the cytosolic malic enzyme to produce NADPH [16, 18], and succinate can directly activate prolyl hydroxylases causing subsequent specific degradation of HIF-1α [19]. In addition, SLA25A10 as well as the oxoglutarate carrier (SLC25A11) are responsible for the import of GSH into the mitochondrial matrix [20]. Since NADPH/NADP^+^ is the principal redox couple involved in intracellular ROS balance and GSH is one of the main antioxidants in the cell, it is expected that the SLC25A10 carrier is important to maintain the redox status, especially in resting cells.

Our data indicate that disturbance of *SLC25A10* expression gives rise to vulnerability to glutamine deprivation in confluent cells and impairs the anchorage independent cell growth. We speculate that confluent *SLC25A10* knockdown cells need glutamine to substitute for the lack of malate from the TCA cycle to produce α-ketoglutarate to be exported to the cytoplasm and there converted to oxaloacetate catalyzed by aspartate aminotransferase and further to malate by malate dehydrogenase (NADP+) for sufficient NADPH production. The increased dependency of glutamine was illustrated by an increased level of glutamate dehydrogenase in *SLC25A10* knockdown cells. The *SLC25A10* knockdown cells were also more sensitive to the anti-cancer drug cisplatin, but not to the nucleoside analog dFdC. Although the mechanism of cisplatin cytotoxicity is complex, cisplatin has been demonstrated to be able to redirect cancer cells from glycolysis to oxidative phosphorylation [21]. The anti-cancer effect of cisplatin was shown to be linked to oxidative stress of tumor cells and an increased level of HIF-1α has been shown to contribute to cisplatin resistance in tumor cells [22, 23]. The nucleoside analogue dFdC is active through incorporation into genomic DNA and thereby the anti-cancer effect is correlated to the rate of DNA replication. Since the knockdown cells had similar growth pattern as mock control cells, the sensitivity to dFdC was not expected to be altered, as was also shown by the results obtained.

The observed cell growth changes can be explained by the altered NADPH production identified in our study. As referred before, NADPH is one of the most important reducing equivalents for both macromolecule synthesis and for scavenging ROS. Cancer cells usually have higher levels of intracellular ROS than normal cells and the relatively high level of ROS in cancer cells may have several effects [24]. To maintain the redox homeostasis and be adjustable to combat oxidative stress are features of special importance for the survival of cancer cells. Metabolism studies with labeled glucose and glutamine have shown that the majority of glucose molecules in proliferating cells were directed into the PPP pathway to produce NADPH and that glutamine was used to replenish mitochondrial TCA intermediates [16]. Other pathways that also can provide NADPH include the methylene tetrahydrofolate dehydrogenases and the malic enzyme pathways. The cytosolic malic enzyme has been predicted to account for nearly 30% of cytosolic NADPH production in dividing cells. Therefore, blocking malate transport might have a more profound effect on NADPH production in resting cells than in dividing cells, especially under stress when the demand of NADPH increase and PPP pathway is down-regulated. Interestingly, our study showed that inhibition of *SLC25A10* led to increased total intracellular NADP levels but with minor effects on NADPH levels. That the *SLC25A10* knockdown cells were deficient in NADPH production was clearly demonstrated by growing the cells in medium without glutamine. We speculate that, although the malate pathway was blocked, the *SLC25A10* knockdown cells grown in normal medium sustained their NADPH levels through a metabolic shift, with increased conversion of pyruvate to acetyl-CoA. This was supported by the increased gene expression level of pyruvate dehydrogenase and decreased inactive form of phosphorylated pyruvate dehydrogenase in the *SLC25A10* knockdown cells. In further support of our hypothesis we showed that supplement of pyruvate could partially rescue cells in medium without glutamine. Consistent with our hypotheisis, we found that HIF-1α was down-regulated and the ROS levels were decreased both in dividing and resting siRNA-SLC cells grown without nutrient limitation. However, upon oxidative stress induced by H_2_O_2_, dividing siRNA-SLC cells produced less ROS than control cells, while the confluent cells had considerably higher ROS levels in comparison to the control cells.

We observed interesting differences in the need for fully active SLC25A10 between proliferating cells and confluent cells. The confluent siRNA-SLC cells were more vulnerable to oxidative stress induced by H_2_O_2_ and to glutamine deprivation than the control cells. Our data showed that the thioredoxin redox system was involved in the process to maintain the redox balance in *SLC25A10* knockdown cells. Although *TXN2* expression decreased in both dividing and confluent cells, the *TXN* and *TXNRD1* genes were up-regulated only in resting cells. Thioredoxin 2 is a small redox protein localized in the mitochondria and plays an important role in the regulation of the mitochondrial membrane potential and protection against oxidant-induced apoptosis. Consistent down-regulation of the *TXN2* gene expression indicated that *SLC25A10* was involved in regulating the mitochondrial redox homeostasis. The increased gene expression levels of *TXN* (the cytosolic form of thioredoxin) and the *TXNRD1* only in resting cells further confirmed the important role of *SLC25A10* for maintaining redox homeostasis in confluent cells.

The gene expression profile of key metabolic enzymes and the decrease of the HIF-1α protein level in siRNA-SLC cells suggested a metabolic shift from glycolysis to mitochondrial oxidative phosphorylation. Although the tumor suppressor p53 expression did not change, the gene expression of its down-stream target p21 decreased. To confirm the involvement of p21 in energy metabolism, we grew untransfected A549 cells in different media conditions with or without pyruvate and glutamine; p21 was only down-regulated in cells growing without both glutamine and pyruvate. Although glutamine deprivation and cisplatin can lead to cell grow arrest, cisplatin is able to induce both, p53 and p21 expression. These results indicate that tumor cells repond to different stress in different ways and also reveal the importance of further studies to investigate the role of p21 in energy metabolism.

In conclusion, our study demonstrates that SLC25A10 is an important carrier in regulating redox homeostasis, especially in resting cells. Inhibition of *SLC25A10* expression can be a strategy to reprogram cell metabolism, compromise cell growth and increase the sensitivity to traditional anticancer drugs such as cisplatin. We suggest SLC25A10 as a novel target to be exploited in the ongoing challenge to combat cancer.

## MATERIALS AND METHODS

### Cell line and reagents

A549 cell line was cultured in DMEM with 10% FBS, 1% penicillin and streptomycin at 37°C, 5% CO_2_. L-glutamine and dialyzed FBS were purchased from Life technologies (Carlsbad, CA). siRNA expression vector p*Silencer*™ Puro Expression Vectors kit was from Applied Biosystems (Life technologies, Carlsbad, CA). RNeasy mini kit was from Qiagen (Hilden, Germany). Primary antibodies Anti-p53 was from Santa Cruz Biotechnology (Dallas, Texas). Anti-p21, anti-HIF-1α, anti-β-actin were from Sigma (St. Louis, MO) and anti-VDAC/porin were from Abcam (Cambridge, UK). Anti-phospho-PDHE1-A type (ser293) was from Millipore (Massachusetts, USA). Anti-SLC25A10 was from Atlas Antibodies (Stockholm, Sweden).

### Establishment of stable *SLC25A10* knockdown cell lines and comparison of cell growth

*SLC25A10* specific siRNA (siRNA-SLC) and mock control siRNA (siRNA-CON) expression plasmids were prepared as described by the manufacturer. Briefly, two specific oligonucleotides were designed using online tools BLOCK-iT™ RNAi designer (http://rnaidesigner.lifetechnologies.com/rnaiexpress/). The siRNA target sequence is 5′-GGATGCAGAACGACGTGAA-3′. The two complimentary oligonucleotides were annealed and ligated into pSilencer 2.1-U6 puro siRNA expression vector and transfected into A549 cells with Fugene 6 HD kit (Promega) (Madison, WI). Stable expression cells were selected with puromycin at 1000 μg/ml, and maintained at 200 μg/ml. The SLC25A10 knock-down clones were screened with real-time PCR (Kapa Biosystems, Wilmington, MA) and the protein expression levels were determine with specific antibody. The clones used for different experiments are indicated in the figures. The growth curves of mock control (siRNA-CON) and *SLC25A10* knockdown (siRNA-SLC) cells were performed by seeding 1.5 × 10^5^ cells in 6-well plates, and cell numbers were counted in triplicate every 3 days for 18 days.

### Mitochondrial staining with mitotracker

For dividing cells, 5 × 10^3^ cells were seeded in 8-well chamber slide (Nunc™ Lab-Tek™ II Chamber Slide™ System, Thermo Scientific, Waltham, MA), and cultured overnight at 37°C and 5% CO_2_. For confluent cells, 1 × 10^4^ cells were placed in 8-well chamber slide, 4 days after the cells reached confluency. Mitochondria were stained with MitoTracker at concentration of 100 ng/ml as described [25]. After staining, cells were fixed with ice-cold methanol for 15 min, permeablized with ice-cold acetone and the nuclei were stained with DAPI as described [26]. 4 areas were randomly chosen from siRNA-SLC and siRNA-CON pictures and total cells and cells with altered mitochondria were counted and the rates were calculated.

### Gene expression profile changes determined with real-time PCR

The mRNA levels of *SLC25A10*, *DRP1*, *MFN1*, *GLUD*, *PDHA1*, *LDHA*, *LDHB*, *TXN*, *TXN2*, *TXNRD1*, *TXNRD2* and *p21* were analyzed by real-time PCR (Kapa Biosystems, Wilmington, MA). The real-time PCR experiments were repeated 3 times independently. Primer sequences for real-time PCR can be found in Supplementary Table S1.

### Measurements of NADP/NADPH and intracellular ROS levels

For NADP/NADPH assay, cell collection and total NADP and NADPH extraction are briefly described below. For dividing cells, 1 × 10^5^ cells were seeded in 3.5 mm dishes and harvested 2 days later. For confluent cells, 3 × 10^5^ cells were placed in 3.5 mm dishes, collected cells 4 days after they reached confluency. For glutamine deprivated cells, 3 × 10^5^ cells were placed in 3.5 mm dishes, when cell reached confluency, the complete medium was replaced with glutamine free medium and continue culture for 4 days. Total NADPt and NADPH levels were analyzed with NADP/NADPH assay kit according to the manufacturer's instruction (Abcam, Cambridge, UK). Total intracellular ROS level was measured as described using 2, 7-dichlorodihydrofluorescein diacetate (CM-H2DCFDA) (Molecular Probes, Carlsbad, CA) as a probe. Briefly 1 × 10^4^ cells were seeded in 96-well plate in DMEM medium. 24 h after plating, the cells were washed twice with HBSS (PBS supplemented with 1.2 mM CaCl_2_ and 10 mM glucose) and incubated with CM-H2DCFDA at final concentration at 10 μM. After 30 min at 37°C, the cells were washed again with HBSS twice and incubated with H_2_O_2_ at 100 μM and HBSS as control for 15 min respectively. The fluorescence was measured by using micro plate fluorimeter with excitation at 495 nm and emission at 530 nm (Varioskan Flash, Thermo Electron Corporation, Waltham, MA).

### Sensitivity to glutamine deprivation experiments

Untransfected, siRNA-CON and siRNA-SLC-2 cells were seeded in 96-well plates at two densities (5 × 10^3^ and 1.5 × 10^4^ cells per well respectively) in 100 μl of complete DMEM medium. After 24 h, the medium from 5 × 10^3^-cell wells was replaced with 100 μl of different conditioned media: complete DMEM, complete DMEM without glutamine, complete DMEM without pyruvate, complete DMEM without pyruvate and glutamine. For the 5 × 10^4^-cell wells, the medium was replaced with different conditioned media as above after 48 h when the cells reached confluency. All media were supplemented with 10% dialyzed FBS and 1% penicillin and streptomycin. The media were replaced every 3 days. Cell viability was determined with Cell Proliferation Kit II (Roche Life Science, Mannhein, Germany). Briefly, 50 μl of XTT labeling reagent and 1 μl of electron coupling reagent was mixed and 50 μl of mixture was added into each well and the plate was incubated in a humidified atmosphere for 3 h, the absorbance was then measured using an ELISA reader (Infinite^®^ M200, Tecan trading AG, Männedorf, Switzerland) at 450 nm with a reference wavelength at 650 nm. The experiments were repeated 3 times. The experiment was also performed with SLC25A10 knock-down Hela cells.

### Soft agar colony assay

Untransfected, siRNA-CON and siRNA-SLC-2 cells were suspended in 0.35% agarose and DMEM supplemented with 10% FBS and 1% penicillin and streptomycin, and seeded over a basal layer of 0.5% agarose. The experiments were set up in 6-well plates at cell density at 5 × 10^3^ cells per well in triplicate. After 4 weeks of culture at 37°C, 5% CO_2_, plates were stained with 0.01% crystal violet for 1 h, and colonies were scored manually from 3 wells for each cell type.

### Sensitivities of tumor cells to anticancer drugs

2 × 10^4^ cells per ml were seeded in 200 μl per well in 96-well micro titer plates in the presence of serial dilutions of the test compounds, 2′, 2′, -difluorodeoxycytidine (dFdC) (Eli Lilly, Indianapolis, IN), and cisplatin (Accord Healthcare, Middlesex, UK). The cells were allowed to proliferate for 48 h before fresh drugs were added. Cell survival was assayed by XTT method after 96 h of drug exposure. The data is presented as the inhibitory concentration (IC_50_), which is defined as the concentration of a drug that is required to inhibit cell proliferation by 50% *in vitro*. Each experiment was performed 3 times.

### Protein levels of SLC25A10, PDH, p53, p21 and HIF-1α in siRNA-SLC cells analyzed by western blot

To measure the SLC25A10, PDH, p53, p21 and HIF-1α protein levels in siRNA-SLC cells, the confluent siRNA-SLC and siRNA-CON cells were harvested and washed with PBS and lyzed in radioimmune precipitation assay buffer (50 mM Tris-HCl, pH 7.6, 150 mM NaCl, 1% Nonidet P40, 0.05% sodium deoxycholate, 0.1% SDS, and protease inhibitors). Western blot was performed using 4–12% precast Bis-Tris gel (NuPAGE) and Amersham Biosciences Hybond-P membrane (GE Healthcare, Pittsburgh). Phosphorylated form of pyruvate dehydrogenase, p53, p21, HIF-1α protein levels were measured with specific primary antibodies, and voltage-dependent anion channel (VDAC) or β-actin was used as loading controls. To determine p53 and p21 protein level changes in different conditioned media, 1.5 × 10^6^ untransfected A549 cells were seeded in 10 mm dishes, after 24 h the medium were replaced with different media as indicated in the legend. The cells were collected after 24 h for Western blot analysis. For cisplatin treatments, 1.5 × 10^6^ cells were seeded in 10 mm dishes in complete DMEM medium, cisplatin was added to the cells at concentration of 10 μM. The cells were cultured for 24 h, and then collected for Western blot as above. The protein expression levels were quantified with Image Studio Lite software (LI-COR Biosciences).

### Statistics

Two-tailed, unpaired Student's *t*-test was used to test for statistical significance of difference in mean values. Significance was set at *p* < 0.05.

## SUPPLEMENTARY FIGURES AND TABLES


