# Supplementary material for: The mitochondrial carrier SLC25A10 regulates cancer cell growth
Source: Oncotarget. 2015 Mar 3;6(11):9271–83. doi: 10.18632/oncotarget.3375 (PMC4496216; doi:10.18632/oncotarget.3375)
Supplement: Supplementary file 1 [file oncotarget-06-9271-s001.pdf]

SUPPLEMENTARY FIGURES AND TABLES

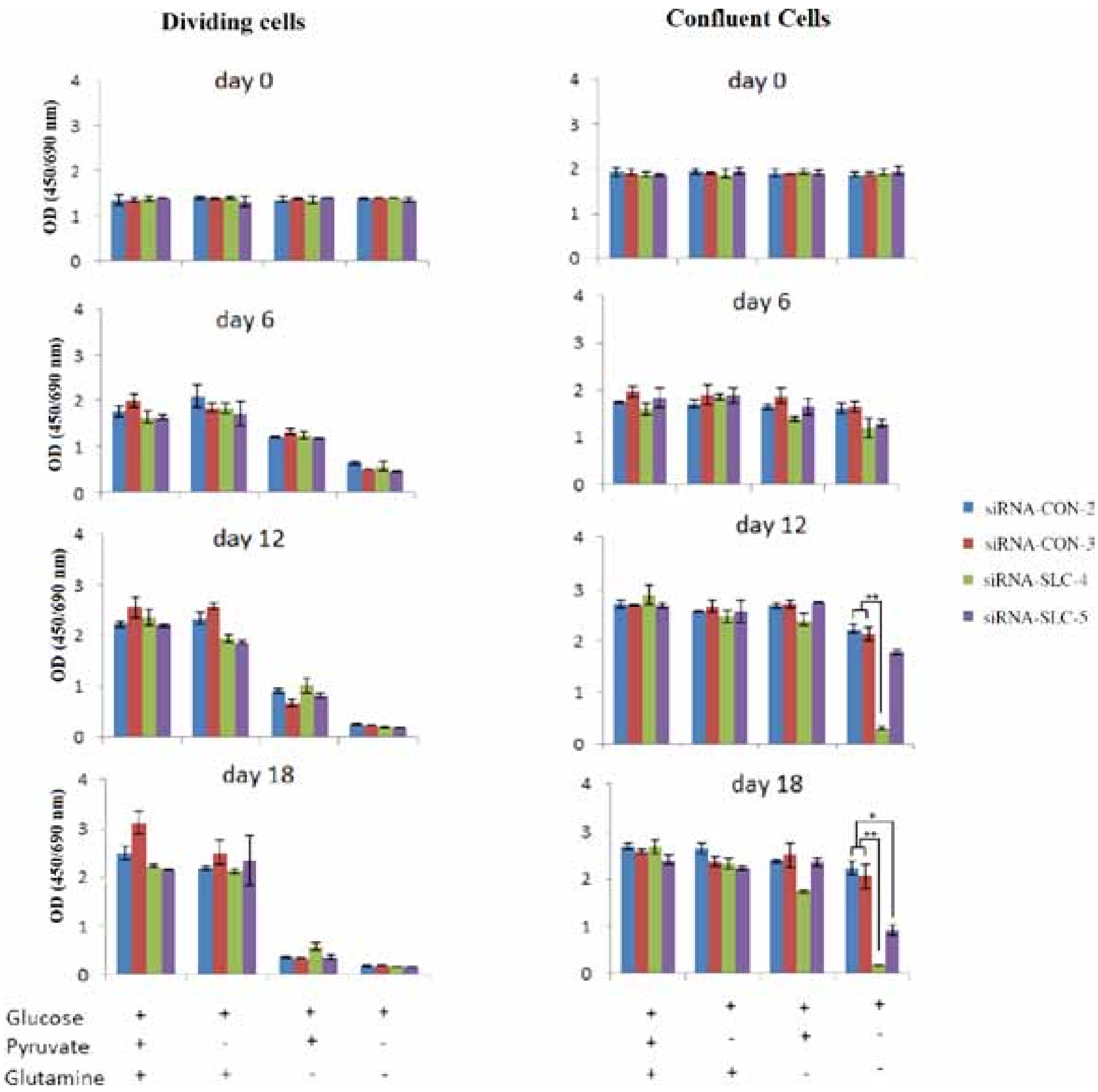

Supplementary Figure S1: SLC25A10 knockdown in A549 cells. Glutamine sensitivity changes in proliferating and confluent cells. Glucose, glutamine and pyruvate concentrations were 25 mM, 2 mM and 1 mM respectively. The experiment was repeated 3 times, and all data are presented as mean ± SD, * represents $p < 0.05$; ** represents $p < 0.01$.

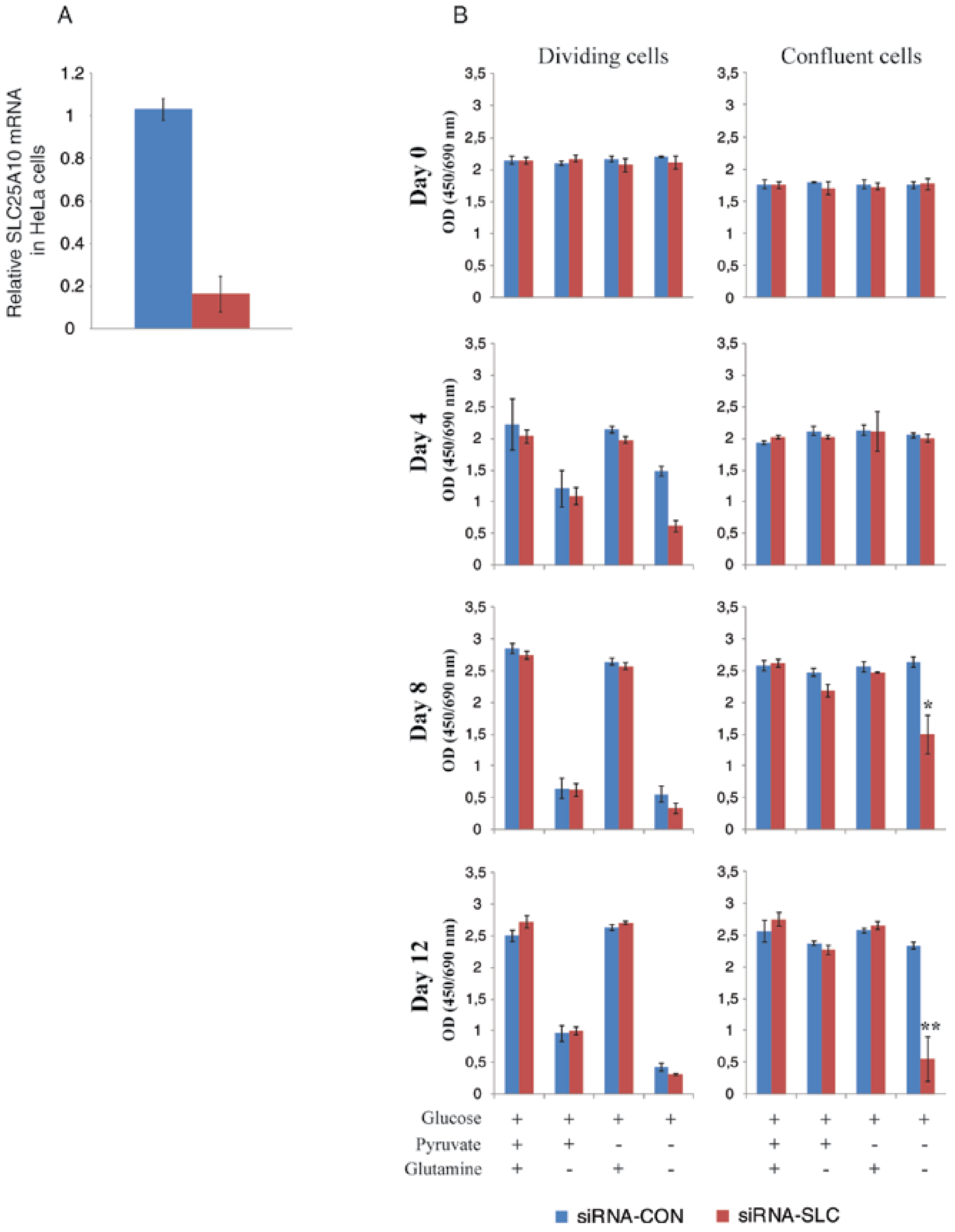

Supplementary Figure S2: SLC25A10 knockdown in HeLa cells. (A) Gene expression of SLC25A10 in siRNA-CON and siRNA-SLC knockdown cells. **(B)** Glutamine sensitivity changes in proliferating and confluent cells. Glucose, glutamine and pyruvate concentrations were 25 mM, 2 mM and 1 mM respectively. The experiment was repeated 3 times, and all data are presented as mean ± SD, * represents $p < 0.05$; ** represents $p < 0.01$.

Supplementary Table S1: Sequences for the primers used in real-time PCR analysis

SLC25A10	5′-CTTAGCACCGGGTACCTCTC-3′<br>5′-GCACATCCACCTGCAATAAA-3′
DRP1	5′-GCTCATTCTTCGGTTCATCA-3′<br>5′-GCGACCATCTGGATCTACCT-3′
MFN1	5′-ACTCCCACTGCTCCTACCAC-3′<br>5′-TCCAAATCACTCCTCCAACA-3′
GLUD1	5′-GACAGGATATCGGGTGCATCT-3′<br>5′-CATGGCTGTGCGCATAATTT-3′
GLUD2	5′-GCCTTGGCATACACAATGGA-3′<br>5′-TGAAGGTCACACCAGCTTCAC-3′
PDHA1	5′-CTGAGAGTGGATGGAATGGA-3′<br>5′-AATAGGCAGCAGCAAACCTT-3′
LDHA	5′-ATGGAGATTCCAGTGTGCCT-3′<br>5′-TGGGTGCAGAGTCTTCAGAG-3′
LDHB	5′-GAGGCAACAGTTCCAAACAA-3′<br>5′-AGCCAGAGACTTTCCCAGAA-3′
CDKN1A<br>(p21)	5′-CAGACCAGCATGACAGATTTC-3′<br>5′-TTAGGGCTTCCTCTTGGAGA-3′
GAPDH	5′-CATCTTCCAGGAGCGAGATCC-3′<br>5′-GCAAATGAGCCCCAGCCT-3′
TXN	5′-CAAGCCTTTCTTTCATTCCC-3′<br>5′-ATGTTGGCATGCATTTGACT-3′
TXNRD1	5′-TGAACAAATTGAAGCAGGGA-3′<br>5′-TGCCAGCATCACCGTATTAT-3′
TXN2	5′-GCCGAGGTTAGAGAAGATGG-3′<br>5′-CACCGCTGACACCTCATACT-3′
TXNRD2	5′-GTGTCCAGCTTCAGGACAGA-3′<br>5′-GCAGAATCTCTTTCCCACCT-3′
GSR	5′-ACTTGCGTGAATGTTGGATG-3′<br>5′-ACCCTCACAACTTGGAAAGC-3′
GSS	5′-AAGCCTATGCTGTGCAGATG-3′<br>5′-TTTGATGGTGCTGGAAAGAG-3′
